# Sjögren’s Syndrome: The Clinical Spectrum of Male Patients

**DOI:** 10.3390/jcm9082620

**Published:** 2020-08-12

**Authors:** Loukas Chatzis, Vasileios C. Pezoulas, Francesco Ferro, Saviana Gandolfo, Valentina Donati, Marco Binutti, Sara Zandonella Callegher, Aliki Venetsanopoulou, Evangelia Zampeli, Maria Mavrommati, Ourania D. Argyropoulou, Giorgos Michalopoulos, Paraskevi V. Voulgari, Themis Exarchos, Chiara Baldini, Fotini N. Skopouli, Dimitrios I. Fotiadis, Salvatore De Vita, Haralampos M. Moutsopoulos, Athanasios G. Tzioufas, Andreas V. Goules

**Affiliations:** 1 Pathophysiology Department, Athens School of Medicine, National and Kapodistrian University of Athens, 11527 Athens, Greece; lukechatzis@gmail.com (L.C.); alikivenetsanopoulou@yahoo.com (A.V.); ariel.argiropoulou@gmail.com (O.D.A.); gdmichalopoulos@gmail.com (G.M.); 2 Unit of Medical Technology and Intelligent Information Systems, University of Ioannina, 45110 Ioannina, Greece; bpezoulas@gmail.com (V.C.P.); dimitris.fotiadis30@gmail.com (D.I.F.); 3 Rheumatology Unit, Department of Clinical and Experimental Medicine, University of Pisa, 56100 Pisa, Italy; francescoferrodoc@gmail.com (F.F.); valentina.donati@gmail.com (V.D.); chiara.baldini74@gmail.com (C.B.); 4 Rheumatology Clinic, Department of Medical area, University of Udine, ASUFC, 33100 Udine, Italy; saviana.gandolfo@uniud.it (S.G.); m.binutti@stdelta.com (M.B.); sarazandonella@gmail.com (S.Z.C.); devita.salvatore@aoud.sanita.fvg.it (S.D.V.); 5 Department of Nutrition and Clinical Dietetics, Harokopio University of Athens, 176 71 Athens, Greece; zampelieva@gmail.com (E.Z.); mmavrommati@euroclinic.gr (M.M.); fskopouli@hua.gr (F.N.S.); hmoutsop@med.uoa.gr (H.M.M.); 6 Rheumatology Clinic, Department of Internal Medicine, Medical School, University of Ioannina, 45110 Ioannina, Greece; pvoulgar@uoi.gr; 7 Department of Informatics, Ionian University, 491 00 Corfu, Greece; themis.exarchos@gmail.com; 8 Department of Biomedical Research, Institute of Molecular Biology and Biotechnology, FORTH, 70013 Ioannina, Greece; 9 Athens Academy of Athens, Chair Medical Sciences/Immunology, 10679 Athens, Greece

**Keywords:** primary Sjögren’s syndrome, male gender, lymphoma

## Abstract

Background: To compare the clinical, serological and histologic features between male and female patients with Sjögren’s syndrome (SS) and explore the potential effect of gender on lymphoma development. Methods: From a multicenter population (Universities of Udine, Pisa and Athens, Harokopion and Ioannina (UPAHI)) consisting of consecutive SS patients fulfilling the 2016 ACR/EULAR criteria, male patients were identified, matched and compared with female controls. Data-driven multivariable logistic regression analysis was applied to identify independent lymphoma-associated factors. Results: From 1987 consecutive SS patients, 96 males and 192 matched female controls were identified and compared. Males had a higher frequency of lymphoma compared to females (18% vs. 5.2%, OR = 3.89, 95% CI: 1.66 to 8.67; *p* = 0.0014) and an increased prevalence of serum anti-La/SSB antibodies (50% vs. 34%, OR = 1.953, 95% CI: 1.19 to 3.25; *p* = 0.0128). No differences were observed in the frequencies of lymphoma predictors between the two genders. Data-driven multivariable logistic regression analysis revealed negative association of the female gender with lymphoma and positive association with lymphadenopathy. Conclusion: Male SS patients carry an increased risk of lymphoma development. Although statistics showed no difference in classical lymphoma predictors compared to females, data-driven analysis revealed gender and lymphadenopathy as independent lymphoma-associated features.

## 1. Introduction

Sjögren’s syndrome (SS) is a complex and heterogeneous disorder that follows a strong trend of female predilection observed in many autoimmune diseases, ranking second after autoimmune thyroiditis [1]. The clinical spectrum of SS extends beyond the exocrine glands, including many extraglandular manifestations, either periepithelial (interstitial nephritis, interstitial lung disease, primary biliary cirrhosis) or extraepithelial B cell mediated manifestations (palpable purpura, peripheral neuropathy, glomerulonephritis), originating from various affected organs and tissues. Non-Hodgkin’s lymphoma of the MALT (mucosa-associated lymphoid tissue) type is a serious complication of the disease with well-established risk factors such as cryoglobulinemia, parotid gland swelling, purpura and low C4 complement levels [2,3,4]. The exact pathogenetic mechanisms underlying the profound female predominance of SS remain unclear. Studies focusing on the roles of the different hormonal milieux between the two genders have so far been inconclusive [5]. Recently, the genetic imbalances created by the different sex chromosomes in each gender have drawn much attention. The presence of a second X chromosome, rich in immune-related genes (*FOXP3, TLR7, CD40LG*, etc.), correlates with the clinical expression and pathogenesis of systemic autoimmune diseases [6]. The unique characteristics of the X chromosome in terms of inactivation, escaping and skewing are implicated in the pathogenesis of systemic autoimmunity, as reflected in the paradigm of males with Klinefelter syndrome (XXY) who exhibit a 36-fold increase in the risk of developing SS than males without (XY) [7]. Therefore, the clinical phenotyping of various subgroups of SS patients including males is of major clinical importance, since it may reflect different underlying pathogenetic mechanisms associated with different clinical expressions, outcomes and responses to treatment.

Literature on the male gender in relation to SS is scarce, involving very low numbers of male patients with heterogeneous classification criteria [8]. In addition, differences in study design and types of data analysis may account for different findings. However, in two recent studies with well-defined classification criteria and a rather small number of male patients, association of the male gender with lymphoma was described [9,10]. Nevertheless, whether male patients with SS have the same clinical phenotypes, laboratory and histological characteristics as well as risk factors for lymphoma development as females is still unknown. In this retrospective study, we aimed to explore the differences in various aspects of SS expression between the two genders with a novel approach, including (i) a well-characterized cohort of males from a multicenter SS population, after careful matching with female controls, and (ii) the use of data-driven approaches to identify lymphoma-associated features.

## 2. Patients and Methods

### 2.1. Study Design

The current study was conducted in the context of the clinical phenotyping of the HarmonicSS project [11]. From a total study population of consecutive SS patients who fulfilled the 2016 ACR/EULAR classification criteria [12] and were followed from May 1984 to March 2019, in five centers from Greece (University of Athens, Harokopio University, University of Ioannina) and Italy (University of Pisa, University of Udine) (UPAHI group), male patients were identified and their cumulative clinical, laboratory and histologic data were collected, integrated and analyzed in a unified dataset, based on the minimum criteria defined by the reference model of the HarmonicSS project [11]. For each male patient, two females were matched according to age of SS onset and disease duration from SS onset until last follow-up, following a maximum of a two-year deviation. SS onset was defined as the year the patient recalled disease-related symptoms began, such as Raynaud’s phenomenon, arthritis, sicca or salivary gland enlargement, while SS diagnosis was used when SS onset could not be specified. Subjective symptoms and systemic organ involvement were based on EULAR Sjögren’s Syndrome Patient Reported Index (ESSPRI) and EULAR Sjögren’s syndrome disease activity index (ESSDAI) definitions respectively [13,14]. Minor salivary gland biopsies, laboratory and objective eye and oral tests were performed in the context of standard of care, according to physicians’ judgment. The two groups were compared on the basis of clinical (dry mouth, dry eyes, parotid gland enlargement, Raynaud’s phenomenon, lymphadenopathy, arthralgias, myalgias, arthritis, palpable purpura, liver involvement, kidney involvement, central and peripheral nervous involvement and lymphoma), laboratory (anti Ro/SSA antibodies, anti La/SSB antibodies, rheumatoid factor, cryoglobulinemia, low C4 complement levels and monoclonal gammopathy) and histologic (focus score and type of lymphoma) features. A subgroup analysis was also conducted to compare Greek and Italian male patients. The study was approved by the local ethical committees of all involved Hospitals and Institutions, after patients’ informed consent and compliance with the General Data Protection Regulation (GDPR). Ethical approval information: University of Athens (National and Kapodistrian University of Athens Bioethics Committee) on 20/07/2017; Harokopio University (Harokopio University Bioethics Committee) on 02/03/2018; Euroclinic hospital (Euroclinic’s hospital Bioethics Committee) on 21/03/2018 ID:111; University of Ioannina (University of Ioannina Medical School bioethics committee) ID:3261:1-2-2019; University of Udine (Unique Ethical Committee of Friuli Venezia Giulia Region) approval code: CEUR-2017-Os-027-ASUIUD; protocol number: 10735, date: 19APR 2017; University of Pisa (Comitato Etico Regionale per la Sperimentazione Clinica della Regione Toscana) Protocol number: 65394.

### 2.2. Statistical Analysis and Data-Driven Approach

Statistical analysis for categorical data was performed by χ^2^ tests, with Yates correction or Fisher’s exact tests when cell counts were < 5 in patients. For numerical data, t-tests or Mann–Whitney tests were used after Shapiro–Wilk normality tests. The fast-correlation-based feature selection (FCBF) algorithm was applied on the unified dataset to identify potentially independent variables for constructing a logistic regression (LR) model with lymphoma as an outcome. The FCBF algorithm is a correlation-based mathematical tool that identifies, among several potentially independent variables, those with the strongest associations with the outcome of interest (e.g., lymphoma) and the weakest associations amongst them, using coefficient similarity as a measure. This subset of FCBF-derived potentially independent variables can be used for the logistic regression model to identify independent lymphoma-associated factors [15]. A conventional 10-fold cross-validation approach was applied to evaluate the sensitivity, specificity and accuracy of the combined FCBF/LR model. Implementation of the FCBF-based multivariable logistic regression approach and statistical analysis were performed using Python 3.6 and GraphPad 7.0a respectively.

## 3. Results

### 3.1. Patient Characteristics

A total of 1987 SS patients fulfilling the 2016 ACR/EULAR criteria were included in the total multicenter population. The number of patients from each of the two Mediterranean countries was similar: Greece *n* = 980, Italy *n* = 1007. Ninety-six (4.8%) males were identified and matched with 192 female controls according to disease duration and age at disease onset. All male patients in the study had normal physical examination results regarding testicle size (length > 3 cm and volume > 20 cc for each testicle) and male phenotypic characteristics (e.g., muscle mass, phallus size, pubic hair, deep voice, absence of gynecomastia) and no family or personal history of infertility. The male-to-female ratio in the total population was approximately 1:20. The median age of males was 50.1 years (range 13–78) while the median age of females was 49.7 years (range 15–78) (Table 1). The median disease duration until last follow-up was six years (range 0–28) for the male group and seven years (range 0–26) for the female group.

### 3.2. Comparison between Males and Females

**Figure 1:**
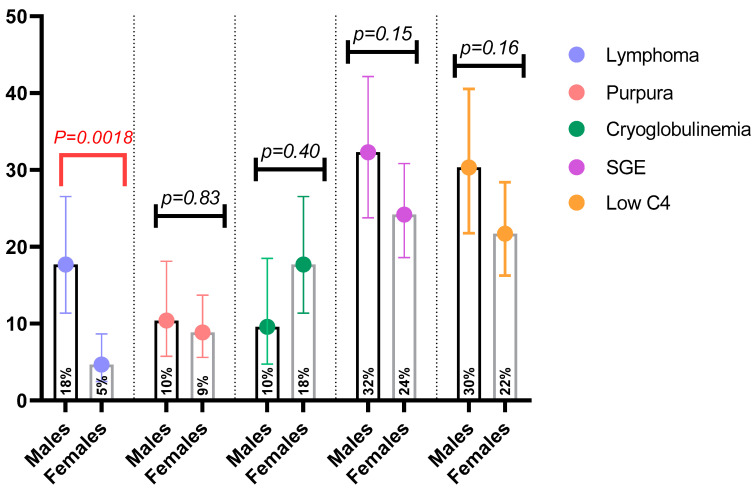
Comparison of lymphoma and classical risk factors between male and female patients with Sjögren’s syndrome.

Clinical manifestations were compared between the two groups (Table 1 and Table 2). Females had a higher frequency of dry mouth compared to males (97.4% vs. 90.6%, OR = 3.84, 95% CI: 1.29 to 10.46; *p* = 0.0266) (Table 1). Dry eyes followed the same trend between males and females, without reaching statistical significance (94% vs. 87% respectively; *p* = 0.07) (Table 1). The prevalence of extraglandular manifestations including non-specific symptoms, liver, pulmonary and renal involvement, as well as B cell associated manifestations such as palpable purpura, glomerulonephritis and peripheral nerve involvement, did not differ significantly between males and females (Table 1 and Table 2, respectively). A statistically significant difference was identified in the frequency of lymphoma among males compared to females (18% vs. 5.2%, OR = 4.89, 95% CI: 1.66 to 8.67; *p* = 0.0014) with no observed differences in the classical risk factors of lymphoma development (Figure 1). To confirm the statistically significant difference in the probability of a male patient presenting with lymphoma, we applied the FCBF algorithm to identify potentially independent variables for lymphoma development on the unified dataset, analyzing 33 distinct features (Table 3). Six variables including lymphadenopathy, salivary gland enlargement, anti-La antibodies, female gender, low C4 and monoclonal gammopathy were identified as potentially independent variables. The multivariable logistic regression model for lymphoma revealed lymphadenopathy as an independent positive risk factor (OR = 6.31, 95% CI: 0.88 to 2.85; *p* = 0.0003) and female gender as an independent negative risk factor (OR = 0.344, 95% CI: −0.31 to −1.92; *p* = 0.01), suggesting positive correlation between male gender and lymphoma development (Table 3). The ROC curve for the FCBF/logistic regression model (FCBF/LR) is presented in Figure S1 (accuracy = 0.92, sensitivity = 0.65, specificity = 0.98, area under the curve (AUC) = 0.87). The histologic types of lymphoma did not differ between the two genders, with MALT lymphoma being the predominant type (Table 2). One male patient developed splenic marginal zone lymphoma and one female had small lymphocytic lymphoma. The frequency of anti-La (SSB) antibodies in the male group compared to females was significantly higher (50% vs. 34%, OR = 1.953, 95% CI = 1.19 to 3.22; *p* = 0.0128), as opposed to anti-Ro (SSA) and rheumatoid factor (Table 1). No differences were observed in focus scores between male and female patients or in the proportion of positive biopsies between the two groups (Table 1). Although the total number of lymphoma patients was small, a comparison between male and female SS lymphoma patients was performed (Table S1). No statistically significant difference was found between the two groups in any clinical, laboratory or histologic parameter. However, male SS patients with lymphoma were older at SS and lymphoma onset, had a longer median disease duration until lymphoma occurrence and presented more frequently with lymphadenopathy (65% vs. 50%), dry skin (27% vs. 0%), serum monoclonality (23% vs. 0%) and cryoglobulinemia (31% vs. 0%) when compared to SS females with lymphoma. The comparison between Greek and Italian male SS patients regarding clinical and serological parameters, classical risk factors of lymphoma, lymphoma or lymphoma histologic subtypes did not reveal differences, except in chronic fatigue, which was higher in Italians (47% for Italians vs. 15% for Greeks; *p* = 0.005).

## 4. Discussion

This was the largest study ever conducted with well-characterized SS male patients who fulfilled the 2016 EULAR/ACR criteria [12] and were compared to matched females, according to disease duration and age at disease onset. Multivariate analysis adjusted for age and follow-up time involving 73 males was performed in only one study; that study showed higher frequency of Raynaud’s phenomenon and thyroiditis only in females, although the number of matched females and follow-up time were not mentioned [16]. In a previous study [10], the fact that both males and females had similar ages at SS onset allowed the two groups to be matched on the basis of the above features, assuming that age and disease duration could potentially affect clinical expression of the disease. The 20:1 female-to-male ratio in our study was considerably different from what has been commonly described in many previous studies, which ranges from 9:1 to 12:1 [17]. The median disease duration is sufficient to allow comparisons regarding clinical phenotype, serology and histology between male and female SS patients. Males exhibit glandular manifestations less frequently but exhibit double positivity of anti-La/SSB plus anti-Ro/SSA autoantibodies more frequently than females, as shown previously [8,10]. In our study, we observed an increased prevalence of lymphoma in SS males compared to females, with no differences in the types of lymphoma or the distribution of traditional lymphoproliferative risk factors between the two genders, suggesting that male gender could be an independent risk factor for lymphoproliferative disorders in SS. The combined FCBF/LR algorithm also confirmed that the male gender had a positive correlation with lymphoma development, since gender was identified as an informative feature.

The higher female-to-male ratio compared to previous studies can be attributed to the lack of consensus criteria for SS in the past and the small number of recruited patients, although recent studies based on more homogeneous cohorts seem to support the higher female-to-male ratio [8,10]. Observed differences in the clinical phenotype relating to a higher frequency of dry mouth and eyes in females are reported for the first time. Given the subjective component of dryness, this may be associated with clinical expression of the disease at a glandular level, the individual’s perception, and the peri-menopausal peak age of SS onset in women, which is probably modified by estrogens and possibly epigenetic regulation of the X chromosome. Regarding anti-La/SSB positivity, it has been proposed that this variable increases the risk of lymphoma [9], and its increased prevalence in males further supports our finding for an association between male gender and lymphoma development. Interestingly, no other differences have been observed among other immunologic parameters including anti-Ro/SSA, RF and cryoglobulins. Anti-La/SSB positivity correlates with more intense inflammatory infiltration of the minor salivary glands, while several reports have shown that ectopic germinal centers within inflamed minor salivary glands are active sites of anti-La/SSB production [18,19,20]. In our study, however, there were no differences in salivary gland focus scores between males and females with FS ≥ 1. Therefore, this finding could be attributed to an anti-idiotypic response directed to anti-La/SSB in female patients, which obscures the anti-La reactivity in these individuals [21].

According to the literature, male gender was associated with lymphoma in SS in only a few studies [9,10,22], and in two of them it was identified as an independent risk factor [9,22]. In both studies, the numbers of lymphomas and recruited male patients were rather small, and there was no appropriate female control matching. Interestingly, the overall proportion of lymphoma among female controls on the unified dataset was 5.2%, which approximates the estimated prevalence of lymphoma among SS cohorts [23,24,25], given that more than 95% of SS patients are female. In the present study, the data-driven multivariable logistic regression analysis highlighted male gender as a lymphoma-associated risk factor. The data-driven FCBF/LR model is considered a novelty. In common practice, selection of potentially independent variables for the logistic regression model is usually based on univariate analysis positive findings and possible biologic associations with the outcome of interest described in the literature, underestimating the independency criterion among proposed variables. Inevitably, this approach carries a risk of bias selection and restricts the pool of potentially independent variables. In our case, application of the FCBF algorithm initially handled and analyzed 33 potentially independent variables in an unbiased manner and used the six strongest (in order of magnitude) to construct the logistic regression model. Interestingly, cryoglobulins and purpura, which are classical risk factors of lymphoma, were not included in the set of potentially independent variables, since the FCBF algorithm on the unified dataset ranked purpura and cryoglobulins in a lower position compared to low C4. This inconsistency could be attributed to the fact that cryoglobulins had a high percentage of missing values on the unified dataset and that low C4 is closely associated with palpable purpura. Although no differences between males and females were observed in terms of classical risk factors of lymphoma including lymphadenopathy, data-driven approaches utilized these variables in a rational way, revealing lymphadenopathy and gender as prominent clinical features to identify high-risk pSS patients who require close follow-up. 

The retrospective nature of our study is an endogenous limitation. Inherent gender bias among systemic autoimmune disorders may result in misdiagnosis and exclude male patients from SS diagnosis, underestimating the clinical diversity of the syndrome and obscuring any gender-related differences. The number of male patients, although this was the largest ever reported, is considered also a limitation, since larger cohorts with more male SS patients and longer disease duration may uncover more phenotypic differences between the two genders. Interestingly, all centers involved are referral centers for SS, managing complicated cases at the national level; therefore, SS cases with milder severity might be underrepresented, especially in males. The absence of karyotyping and sex hormone measurements could also be considered a limitation, but such studies are pending. To better understand the differences in various aspects of the disease between the two genders, other studies are required focusing not only on the hormonal state of patients but also on the roles of the X and Y chromosomes that may shape and define the clinical phenotype of the disease by regulating the expression of the complex gene network implicated in pathogenesis. Despite the above-mentioned limitations, this study highlights the potentialities of data-driven approaches in refining patient gender differences, opening new avenues for investigation of the underlying biological basis of these differences as a prerequisite towards precision medicine in autoimmunity. Phenotypic differences among SS patients are most likely defined by several factors such as age, gender, race and ethnic group. In addition, environmental factors can trigger the initial autoimmune response, e.g., a viral infection may also affect phenotypic expression of the disease. Single cohorts cannot reveal such differences because of the small number of patients. This limitation can be overcome only by a multicenter SS population. Large data gathering and employment of data-mining tools may provide the opportunity to study in more detail the effect on phenotype of more than one factor (e.g., gender and young age at SS onset), creating distinct clinical clusters.

## Figures and Tables

**Table 1:** Comparison of glandular, extraglandular, serological and histological features between male and female patients with Sjögren’s syndrome (female control population was matched in a 1:2 ratio based on SS age onset and disease duration).

FEATURE	Females (*n* = 192)	Males (*n* = 96)	*p*-Value
**BASIC CHARACTERISTICS**			
Age onset of first symptom	49.7	50.1	0.7928
Median disease duration until last follow-up (years)	7 (0–26)	6 (0–28)	0.2450
Greek patients	933	47	
Italian patients	958	49	
**GLANDULAR MANIFESTATIONS**			
Dry mouth	97% (186/191)	91% (87/96)	**0.0266**
Dry eyes	94% (180/191)	87% (83/95)	0.0747
Salivary gland enlargement	24%(45/186)	32% (31/96)	0.1900
Dry skin	9%(14/148)	7% (5/74)	0.6715
**EXTRAGLANDULAR MANIFESTATIONS**			
Chronic Fatigue	38% (57/148)	27% (21/77)	0.1733
Raynaud’s phenomenon	26% (48/185)	19% (18/94)	0.3290
Arthritis	9% (16/185)	10% (10/95)	0.7679
Arthralgia	52% (99/192)	47% (45/95)	0.5320
Myositis	0.5% (1/192)	2% (2/96)	0.2585
Interstitial renal disease	1% (2/190)	0% (0/95)	0.5540
Primary biliary cirrhosis	0% (0/192)	2% (2/96)	0.1103
Autoimmune hepatitis	0% (0/192)	2% (2/95)	0.1103
Interstitial lung disease	5% (9/192)	8% (8/96)	0.3309
**SEROLOGY**			
Rheumatoid Factor	47% (84/177)	51% (45/89)	0.7278
Anti-Ro	72% (136/190)	73% (69/95)	0.9628
Anti-La	34% (64/189)	50% (47/94)	**0.0128**
LOW C4	22% (38/175)	30% (27/89)	0.1657
Monoclonality	4% (7/192)	7% (7/96)	0.2866
Cryoglobulinemia	6% (8/135)	10% (7/73)	0.4877
**HISTOLOGY**			
Focus score ≥ 1	80% (90/113)	78% (42/54)	0.5425
Median FS in positive biopsies	1.383	1.477	0.2786

Bold text indicates a statistically significant difference with a *p*-value less than 0.05.

**Table 2:** Comparison of lymphoma and B cell associated manifestations between male and female patients with Sjögren’s syndrome (female control population was matched in a 1:2 ratio based on SS age onset and disease duration).

FEATURE	Females (*n* = 192)	Males (*n* = 96)	*p*-Value
Palpable purpura	9% (17/192)	10% (10/96)	0.8302
Vasculitic ulcer	2% (3/192)	1% (1/96)	1
Glomerulonephritis	0.5% (1/191)	3% (3/96)	0.1106
Peripheral nerve involvement	2% (4/192)	3% (3/96)	0.6895
Lymphadenopathy	9% (18/192)	17% (16/95)	0.0994
Central nervous system involvement	2% (3/192)	2% (2/96)	1
**Lymphoma**	5% (10/191)	18% (17/96)	**0.0013**
MALT	80% (8/10)	76% (13/17)	0.99
DLBCL	10% (1/10)	18% (3/17)	0.99

Bold text indicates a statistically significant difference with a *p*-value less than 0.05.

**Table 3:** FCBF-based multivariable logistic regression analysis for investigating the effect of gender on lymphoma development.

Prominent Feature *	Regression Coefficient	Odds Ratio	*p*-Value	CI Low	CI Upper
**Lymphadenopathy**	1.869	6.549	0.0003 **	2.456	17.482
**SGE**	0.689	2.006	0.129	0.853	4.724
**Anti-La**	0.682	1.989	0.11	0.884	4.477
**Female Gender**	−1.119	0.332	0.011 **	0.148	0.742
**Low C4 (<20 mg/dl)**	0.465	1.599	0.337	0.629	4.069
**Monoclonal gammopathy**	0.537	1.728	0.512	0.353	8.592

* The strongest potentially independent variables identified by the fast-correlation-based feature selection (FCBF) algorithm to build the logistic regression model, after analyzing the following initial features included in the dataset: ethnicity, gender, disease duration (onset), dry mouth, dry eyes, anti-Ro, anti-La, Rheumatoid Factor, focus score, germinal centers in biopsy, monoclonal gammopathy, SGE (Salivary Gland Enlargement), lymphadenopathy, low C4, dry skin, chronic fatigue, arthralgias-myalgias, arthritisis, Raynaud’s phenomenon, palpable purpura, vasculitic ulcer, myositis, peripheral neuropathy, CNS involvement, liver-autoimmune hepatitis, liver-PBC (Primary Biliary Cirrhosis), interstitial lung disease, interstitial renal disease, kidney/glomerulonephritis, heart valvular disease, cryoglobulinemia, and lymphoma. ** < 0.05 (95% confidence interval): independent lymphoma-associated features revealed by the logistic regression model.

## Supplementary Materials

The following are available online at https://www.mdpi.com/2077-0383/9/8/2620/s1, Figure S1: Performance of the combined FCBF/logistic regression model with lymphoma as an outcome, after incorporating both males’ and females’ data (10-fold cross validation approach), Table S1: Comparison of clinical, serological, and histologic features between male and female patients with lymphoma developed in the context of Sjogren Syndrome.
