# Supplementary material for: Sjögren’s Syndrome: The Clinical Spectrum of Male Patients
Source: J Clin Med. 2020 Aug 12;9(8):2620. doi: 10.3390/jcm9082620 (PMC7463756; doi:10.3390/jcm9082620)
Supplement: Supplementary file 1 [file jcm-09-02620-s001.pdf]

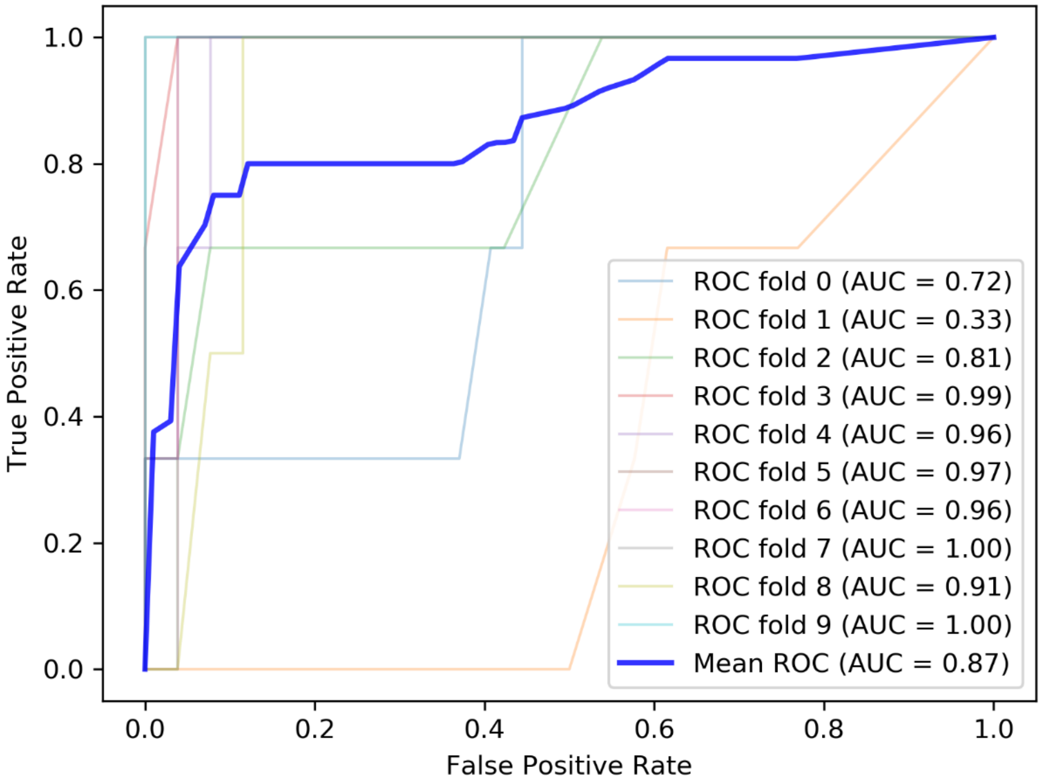

Figure S1. Performance of the combined FCBF/logistic regression model with lymphoma as an outcome, after incorporating both males' and females' data (10-fold cross validation approach). Accuracy = 0.92, sensitivity = 0.65, specificity = 0.98, Area under the curve (AUC) = 0.87.

Table S1. Comparison of clinical, serological, and histologic features between male and female patients with lymphoma developed in the context of Sjogren Syndrome.

**Supplementary Table 1**	**MALES *n*=17**	**FEMALES n=10**	***P* VALUE**
Median disease duration from Sjogren onset to lymphoma (range)	5 (0–26)	3 (0–23)	0.2664
Median age at Sjogren's onset (range)	50 (32–72)	46 (19–61)	0.2475
Median age at lymphoma diagnosis (range)	57(35–75)	52(32–65)	0.2570
Salivary gland enlargement *n, (%)*	9/17 (53%)	7/10 (70%)	0.4475
Lymphadenopathy *n, (%)*	11/17 (65%)	5/10 (50%)	0.7297
Palpable purpura *n, (%)*	4/17 (24%)	2/10 (20%)	0.9999
Low C4 *n, (%)*	8/15 (53%)	5/10 (50%)	0.8063
Cryoglobulinemia *n (%)*	4/13 (31%)	0/8 (0%)	0.1312
Monoclonality *n (%)*	4/17 (23%)	0/10 (0%)	0.2638
Rheumatoid factor *n (%)*	11/16 (69%)	8/10 (80%)	0.6680
Anti-Ro antibody *n (%)*	14/17 (82%)	9/10 (90%)	0.9999
Anti-La antibody *n (%)*	12/17 (71%)	8/10 (80%)	0.6784
Dry mouth *n (%)*	17/17 (100%)	10/10 100%)	0.9999
Dry eyes *n (%)*	17/17 (100%)	10/10 100%)	0.9999
Dry skin *n (%)*	3/11 (27%)	0/7 (0%)	0.2451
Chronic fatigue *n (%)*	5/11 (45%)	3/7 (45%)	0.9999
Arthralgias *n (%)*	8/17 (47%)	4/10 (40%)	0.9999
Arthritis *n (%)*	2/17 (12%)	1/10 (10%)	0.9999
Raynaud *n (%)*	4/17 (24%)	2/10 20%)	0.9999
Lung involvement *n (%)*	1/17 (6%)	2/10 (20%)	0.5350
Kidney involvement *n (%)*	0/17 (0%)	0/10 (0%)	0.9999
Liver involvement *n (%)*	0/17 (0%)	0/10 (0%)	0.9999
Nervous system involvement *n (%)*	1/17 (6%)	0/10 (0%)	0.9999
Focus score ≥1 *n (%)*	8/10 (80%)	5/7 (71%)	0.9999
